# Measuring the effectiveness of integrated vector management with targeted outdoor residual spraying and autodissemination devices on the incidence of dengue in urban Malaysia in the iDEM trial (intervention for Dengue Epidemiology in Malaysia): study protocol for a cluster randomized controlled trial

**DOI:** 10.1186/s13063-021-05298-2

**Published:** 2021-05-30

**Authors:** Mitra Saadatian-Elahi, Neal Alexander, Tim Möhlmann, Carole Langlois-Jacques, Remco Suer, Nazni Wasi Ahmad, Rose Nani Mudin, Farah Diana Ariffin, Frederic Baur, Frederic Schmitt, Jason H. Richardson, Muriel Rabilloud, Nurulhusna Ab Hamid

**Affiliations:** 1 Service Hygiène, Epidémiologie, Infection, Vigilance et Prévention, Centre Hospitalier Edouard Herriot, Hospices Civils de Lyon, Lyon, France; 2 CIRI, Centre International de Recherche en Infectiologie, (Equipe Laboratoire des Pathogènes Emergents), Univ Lyon, Inserm, U1111, Université Claude Bernard Lyon 1, CNRS, UMR5308, ENS de Lyon, F-69007 Lyon, France; 3 MRC Tropical Epidemiology Group, Department of Infectious Disease Epidemiology, London School of Hygiene and Tropical Medicine, Keppel St, London, WC1E 7HT UK; 4 In2Care B.V., Marijkeweg 22, 6709PG Wageningen, The Netherlands; 5 Université de Lyon, F-69000, Lyon, France; Université Lyon 1, F-69100, Villeurbanne, France; Hospices Civils de Lyon, Pôle Santé Publique, Service de Biostatistique et Bioinformatique, F-69003, Lyon, France; CNRS, UMR 5558, Laboratoire de Biométrie et Biologie Évolutive, Équipe Biostatistique-Santé, F-69100 Villeurbanne, France; 6 Medical Entomology Unit, WHO Collaborating Centre for Vectors, Institute for Medical Research, Ministry of Health Malaysia, National Institutes of Health, Block C, Jalan Setia Murni U13/52, Seksyen U13, Setia Alam, 40170 Shah Alam, Malaysia; 7 Vector Borne Disease Sector, Disease Control Division, Ministry of Health Malaysia, Level 4, Block E10, Complex E, Federal Government Administrative Center, 62590 Putrajaya, Malaysia; 8 Bayer S.A.S, Environnemental Science, Crop Science Division, 16 rue Jean Marie Leclair, 69266 Lyon, Cedex 09 France; 9 Innovative Vector Control Consortium, Pembroke Place, L3 5QA, Liverpool, UK

**Keywords:** *Aedes*, Dengue, Malaysia, Epidemiology, Cluster Randomized, Vector control, Autodissemination, outdoor residual spray

## Abstract

**Background:**

In common with many South East Asian countries, Malaysia is endemic for dengue. Dengue control in Malaysia is currently based on reactive vector management within 24 h of a dengue case being reported. Preventive rather than reactive vector control approaches, with combined interventions, are expected to improve the cost-effectiveness of dengue control programs. The principal objective of this cluster randomized controlled trial is to quantify the effectiveness of a preventive integrated vector management (IVM) strategy on the incidence of dengue as compared to routine vector control efforts.

**Methods:**

The trial is conducted in randomly allocated clusters of low- and medium-cost housing located in the Federal Territory of Kuala Lumpur and Putrajaya. The IVM approach combines: targeted outdoor residual spraying with K-Othrine Polyzone, deployment of mosquito traps as auto-dissemination devices, and community engagement activities. The trial includes 300 clusters randomly allocated in a 1:1 ratio. The clusters receive either the preventive IVM in addition to the routine vector control activities or the routine vector control activities only. Epidemiological data from monthly confirmed dengue cases during the study period will be obtained from the Vector Borne Disease Sector, Malaysian Ministry of Health e-Dengue surveillance system. Entomological surveillance data will be collected in 12 clusters randomly selected from each arm.

To measure the effectiveness of the IVM approach on dengue incidence, a negative binomial regression model will be used to compare the incidence between control and intervention clusters. To quantify the effect of the interventions on the main entomological outcome, ovitrap index, a modified ordinary least squares regression model using a robust standard error estimator will be used.

**Discussion:**

Considering the ongoing expansion of dengue burden in Malaysia, setting up proactive control strategies is critical. Despite some limitations of the trial such as the use of passive surveillance to identify cases, the results will be informative for a better understanding of effectiveness of proactive IVM approach in the control of dengue. Evidence from this trial may help justify investment in preventive IVM approaches as preferred to reactive case management strategies.

**Trial registration:**

ISRCTN ISRCTN81915073. Retrospectively registered on 17 April 2020.

**Supplementary Information:**

The online version contains supplementary material available at 10.1186/s13063-021-05298-2.

## Administrative information

Note: the numbers in curly brackets in this protocol refer to SPIRIT checklist item numbers. The order of the items has been modified to group similar items (see http://www.equator-network.org/reporting-guidelines/spirit-2013-statement-defining-standard-protocol-items-for-clinical-trials/).

**Table:** 

Title {1}	Measuring the effectiveness of integrated vector management with targeted outdoor residual spraying and autodissemination devices on the incidence of dengue in urban Malaysia in the iDEM trial (intervention for Dengue Epidemiology in Malaysia): Study protocol for a cluster randomized controlled trial Acronym: Intervention for Dengue Epidemiology in Malaysia (iDEM)
Trial registration {2a and 2b}.	ISRCTN-81915073, registered on 17 April 2020
Protocol version {3}	Version 01, October 2020
Funding {4}	Malaysia Ministry of Health (Malaysia), Bayer SAS Environmental Science, Crop Science Division (France), UK Aid from the UK government in a grant administrated through Innovative Vector Control Consortium (IVCC, UK), Fondation innovation en Infectiologie (FINOVI, France)
Author details {5a}	^1^Service Hygiène, Epidémiologie, Infection, Vigilance et Prévention, Centre Hospitalier Edouard Herriot, Hospices Civils de Lyon, Lyon, France ^2^CIRI, Centre International de Recherche en Infectiologie, (Equipe Laboratoire des Pathogènes Emergents), Univ Lyon, Inserm, U1111, Université Claude Bernard Lyon 1, CNRS, UMR5308, ENS de Lyon, F-69007, Lyon, France. ^3^MRC Tropical Epidemiology Group, Department of Infectious Disease Epidemiology, London School of Hygiene and Tropical Medicine, Keppel St, London, WC1E 7HT. ^4^In2Care B.V., Marijkeweg 22, 6709PG Wageningen, the Netherlands ^5^Université de Lyon, F-69000, Lyon, France; Université Lyon 1, F-69100, Villeurbanne, France; Hospices Civils de Lyon, Pôle Santé Publique, Service de Biostatistique et Bioinformatique, F-69003, Lyon, France; CNRS, UMR 5558, Laboratoire de Biométrie et Biologie Évolutive, Équipe Biostatistique-Santé, F-69100, Villeurbanne. ^6^ Medical Entomology Unit, WHO Collaborating Centre for Vectors, Institute for Medical Research, Ministry of Health Malaysia, National Institutes of Health, Block C, Jalan Setia Murni U13/52, Seksyen U13, Setia Alam, 40170, Shah Alam, Malaysia. ^7^Vector Borne Disease Sector, Disease Control Division, Ministry of Health Malaysia, Level 4, Block E10, Complex E, Federal Government Administrative Center, 62590 Putrajaya, Malaysia ^8^Bayer S.A.S, Environmental Science, Crop Science Division; 16 rue Jean Marie Leclair ; 69266 Lyon Cedex 09 ; France ^9^IVCC, Pembroke Place, L3 5QA, Liverpool, UK
Name and contact information for the trial sponsor {5b}	Dr Haji Tahir Bin Iris Institute for Medical Research (IMR), Ministry of Health Malaysia, National Institutes of Health, Block C, Jalan Setia Murni U13/52, Seksyen U13, Setia Alam, 40170, Shah Alam, Malaysia
Role of sponsor {5c}	As sponsor of the trial, IMR assumes responsibilities under the agreement outlined in the “Consortium and Collaboration Agreement” signed by all collaborators and funders. FB and FS from Bayer were involved in the writing and publication of the report.

## Background

Dengue fever, caused by one of the four dengue virus serotypes (DENV1-DENV4), is a mosquito-borne disease of public health importance due to its increasing burden worldwide [1]. With 390 million estimated infections and 10,000 deaths per year, dengue is the predominant mosquito-borne virus in humans [2]. The threat of a possible dengue outbreak due to the introduction of dengue virus by viremic travellers into areas where *Aedes albopictus* mosquitoes have become established is also of concern. Global warming may further exacerbate this situation by enabling greater spread and transmission in more temperate regions of the world such as Europe [1]. In Malaysia, *Aedes aegypti* and *Aedes albopictus* are the primary and secondary mosquito species responsible for dengue transmission [3]. The incidence of dengue in Malaysia increased from 31.6 reported cases per 100,000 inhabitants in the year 2000 to 159.7 in 2010 [4] and to 396.4 per 100,000 inhabitants in 2015 [5]. In 2010, the country spent an estimated US$73.5 million (95% confidence interval CI = US$62.0–US$86.3 million) on the national dengue vector control programme [6]. The control of dengue in Malaysia is based on rapid reactive vector management within 24 h of a dengue case being reported. The programme consists of space spray, source reduction, and larviciding. Space spray has however a relatively short life span compared to residual spraying [7, 8], and repeated use in a non-targeted application increases the potential of mosquitoes to develop resistance.

Vector control has been shown to be highly effective in reducing the density of *Aedes* populations [9], but there is limited evidence of its efficacy in reducing the incidence of *Aedes*-borne diseases [10, 11]. Designing more effective and preventive rather than reactive vector control programmes with combined interventions could be a key solution in dengue prevention and control. In the absence of an effective dengue vaccine, the World Health Organization (WHO) calls for locally adapted and sustainable integrated vector management (IVM) [12, 13].

The study protocol presented here is a cluster randomized controlled trial (cRCT) based on proactive IVM that combines three components: (1) targeted outdoor residual spraying (TORS) with K-Othrine Polyzone (Bayer SAS), (2) deployment of mosquito traps as auto-dissemination devices (ADDs, In2Care® Mosquito Traps, In2Care B.V., The Netherlands), and (3) community engagement (CE) activities.

K-Othrine® Polyzone, with deltamethrin as its active ingredient (62.5 g/L), has been approved for use by the WHO [14] and prequalified as vector control product (PQT-VC reference 008-004).

Auto-dissemination devices are black plastic containers that mimic breeding sites for female mosquitoes [15]. These user-friendly control devices target cryptic, hard to find breeding sites and facilitate precision-targeted larval control and continuous breeding suppression of vector populations [15, 16].

Previous field evaluation of TORS and ADDs showed effective reduction of the mosquito population [3, 17]. In addition, the combination of insecticides with different modes of action in the same programme is likely to limit insecticide resistance in the local *Aedes* vector population. However, to the best of our knowledge, the combination of these components has not yet been tested in a large trial with dengue incidence as the primary endpoint.

Local communities are key actors in vector control programmes and their engagement is essential for success and sustainability of such programmes [18–20]. We expect that community engagement by proactive communication and information-sharing with the population of the study areas will increase their adherence to operational procedures, thus enhancing the efficacy of the proposed combined vector control tools to promote favourable outcomes with respect to decreased dengue incidence.

## Objectives

The principal objective of this cRCT trial is to quantify the effectiveness of a preventive IVM strategy combining TORS, deployment of ADDs, and CE activities on the incidence of dengue in randomly selected areas in the Federal Territory of Kuala Lumpur and Putrajaya (Malaysia) as compared to routine vector control efforts.

The secondary objective is to evaluate the effectiveness of the IVM approach on the population density of *Aedes* mosquitoes.

## Trial design

The study is a non-blinded superiority cRCT conducted in selected areas of the Federal Territory of Kuala Lumpur and Putrajaya, Malaysia. The trial protocol was registered with the ISRCTN registry on 17 April 2020 as ISRCTN81915073.

For the purpose of the trial, a cluster is defined as a locality, i.e. one residential area composed of neighbouring buildings that share the same facilities such as parking lots, food court, groceries store, playground, and community halls.

The trial includes 300 clusters (mean number of buildings per clusters = 4) which were randomly allocated in a 1:1 ratio, to receive either the proactive IVM in addition to the routine vector control activities or the routine vector control activities only (see the “Interventions” section for more details). The overall population size in the control and intervention arms are both estimated around 432.000 individuals.

## Methods

### Study setting

**Fig. 1:**
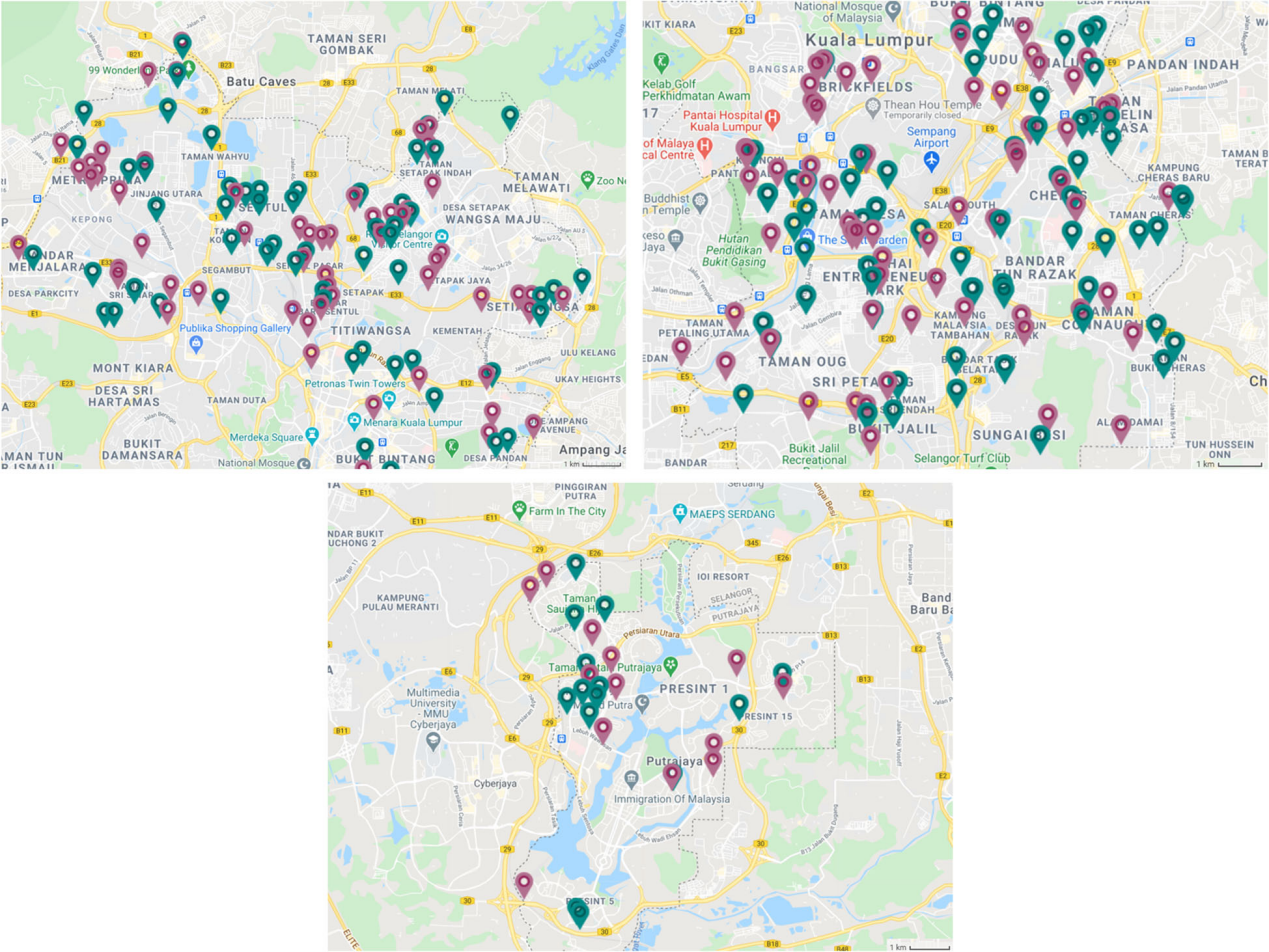
Map of the study areas: Federal Territory of Kuala Lumpur and Putrajaya

The trial is conducted in selected clusters of low- and medium-cost housing located in the Federal Territory of Kuala Lumpur and Putrajaya, Malaysia (Fig. 1). The definition of low and medium cost is based on the average price of an apartment in a given area. Low-cost building is defined as a flat/apartment with the price of less than 60 K€ while the price of medium-cost housing is more than 60 K€ but less than 100 K€. Kuala Lumpur covers 243km^2^ (94 square miles) and had an estimated population of 1.77 million in 2020. Putrajaya covers 49 km^2^ (18.9 square miles) and had an estimated population of 0.10 million in 2020.

Localities (clusters) in the Federal Territory of Kuala Lumpur and Putrajaya with recurrent dengue outbreaks reported from 2015 to 2018 were eligible (*n* = 895). We excluded localities with missing data on the population (*n* = 102), localities within a 1 km radius of an ongoing study with *Wolbachia* (*n* = 3), high-cost localities (*n* = 246), and localities with less than 1000, or more than 8000 inhabitants (*n* = 215). Of the remaining 329 localities, 300 with the highest incidence rates in 2015–2016 were retained for the trial. Eligibility criteria for a dengue case to be registered in the e-Dengue surveillance system is provided (Additional file 1). Only confirmed dengue cases will be included (see the “Outcomes” section).

### Interventions

**Fig. 2:**
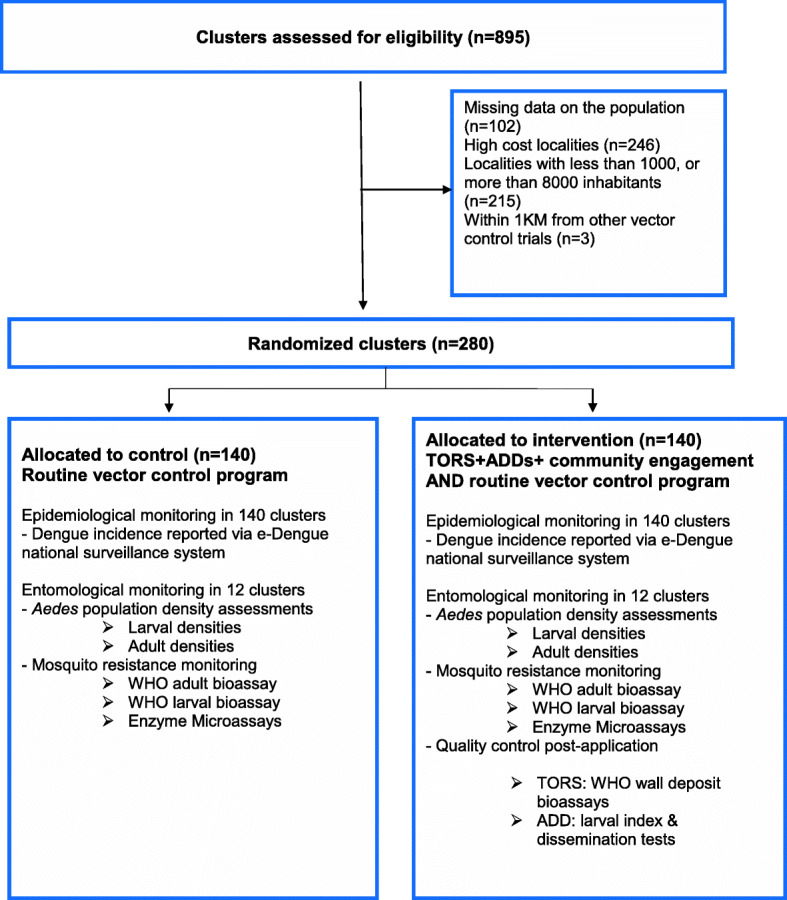
Flowchart of cluster selection and allocation, Federal Territory of Kuala Lumpur and Putrajaya

The study flowchart for selection and randomization of eligible clusters is shown in Fig. 2. Clusters (localities) randomly selected to the intervention arm will receive the proactive IVM strategy, i.e. TORS, deployment of ADDs (performed by the staff of a pest control organisation hired for the purpose of the trial), and active community engagement. Routine dengue prevention and control measures are under the responsibility of the Malaysian Ministry of Health and should be maintained in both control and intervention clusters as per Standard Operational Procedures and guidelines given by the Malaysian Ministry of Health. This programme consists of health education, space spray (thermal fogging and ultra-low volume space spray), source reduction, and larviciding within 24 h a dengue case is being reported. All information on the routine insecticide and technical information (date, extent of activity, place, etc.) is recorded and described in trial reports.

The trial will run for 2 years and the time schedule of enrolment, intervention and data monitoring activities is shown in Table 1.

**Table 1:** Time schedule of intervention, data collection, and analysis

Year	2019		2020												2021												2022												
Project activities	Nov	Dec	Jan	Feb	Mar	Apr	May	Jun	Jul	Aug	Sep	Oct	Nov	Dec	Jan	Feb	Mar	Apr	May	Jun	Jul	Aug	Sep	Oct	Nov	Dec	Jan	Feb	Mar		Apr	May	Jun	Jul	Aug	Sep		Oct	
**Community engagement**																																							
Site visit and site scouting	X	X	X	X	X	X	X	X	X																														
Community engagement	X	X	X	X	X	X	X	X	X	X	X	X	X	X	X	X	X	X	X	X	X	X	X	X	X	X	X	X	X		X	X	X	X	X				
**Vector control activities**																																							
ORS—cycle 1				X	X	X	X	X	X	X	X																												
ORS—cycle 2								X	X	X	X	X	X	X	X																								
ORS—cycle 3												X	X	X	X	X	X	X	X																				
ORS—cycle 4															X	X	X	X	X	X	X	X	X																
ORS—cycle 5																			X	X	X	X	X	X	X	X	X												
ORS—cycle 6																							X	X	X	X	X	X	X		X	X	·						
ADDs distribution				X	X	X	X	X	X	X	X																												
ADDs service						X	X	X	X	X	X	X	X																										
ADDs service								X	X	X	X	X	X	X	X																								
ADDs service										X	X	X	X	X	X	X	X																						
ADDs service												X	X	X	X	X	X	X	X																				
ADDs I service														X	X	X	X	X	X	X	X																		
ADDs service															X	X	X	X	X	X	X	X	X																
ADDs service																	X	X	X	X	X	X	X	X	X														
ADDs service																			X	X	X	X	X	X	X	X	X												
ADDs service																						X	X	X	X	X	X	X	X										
ADDs Service																							X	X	X	X	X	X	X		X	X							
ADDs service																										X	X	X	X		X	X	X	X					
ADDS collection																																				x		·	
**Entomology endpoints**																																							
Ovitrapping				X	X	X	X	X	X	X	X	X	X	X	X	X	X	X	X	X	X	X	X	X	X	X	X	X	X	X	X	X	X						
Larvae identification of the larvae				X	X	X	X	X	X	X	X	X	X	X	X	X	X	X	X	X	X	X	X	X	X	X	X	X	X	X	X	X	X						
Mosquito colonization				X	X	X	X	X	X	X	X	X	X	X	X	X	X	X	X	X	X	X	X	X	X	X	X	X	X	X	X	X	X						
Larval survey				X	X	X	X	X	X	X	X	X	X	X	X	X	X	X	X	X	X	X	X	X	X	X	X	X	X	X	X	X	X						
Adult survey				X	X	X	X	X	X	X	X	X	X	X	X	X	X	X	X	X	X	X	X	X	X	X	X	X	X	X	X	X	X						
Determination of mosquito resistance		X	X	X	X	X	·														X													X					
WHO wall deposit bioassay					X	X	X	X	X	X	X	X	X	X	X	X	X	·																					
**Epidemiology endpoints**																																							
Collection of reported dengue cases (e-Dengue)				X	X	X	X	X	X	X	X	X	X	X	X	X	X	X	X	X	X	X	X	X	X	X	X	X	X	X	X	X	X	X	X	X	X		
Data analysis and publication																																		X	X	X	X	X	X

#### Targeted outdoor residual spraying

K-Othrine Polyzone is a polymer enhanced suspension concentrate aqueous formulation which has low odour and high level of safety. The polymer technology used in this formulation protects the active ingredients from weather conditions, rainfall, and mechanical abruption and ensures they remain available on treated surfaces for up to 3 months, thereby reducing environmental contamination. The insecticide dosage is 25 mg/m^2^ and is applied by trained operators using a residual hand compression sprayer (Semco, Japan). The target spray areas are outer walls covered or partly covered (corridors, staircase, parking, lobby or any corner liable to become a resting spot for mosquitoes) in all floors. We exclude sections of wall close to power plugs, elevator buttons, walls covered with wall paper, oil-based painted walls, or tiles. Residents are requested to remove edible plants, hanging clothes, pet cages (e.g. cats, dogs and birds), and to cover aquaria during the day of the intervention. Parts of corridors with such objects are not sprayed. Based on our previous findings showing the residual bio-efficacy of K-Othrine® Polyzone for 16 weeks [17, 21], TORS is conducted every 4 months in all premises in al intervention areas.

To control the quality of TORS spraying, each spray pump is equipped with a control flow valve. The latter is a device that fits next to the nozzle and ensures that output remains constant as the pressure in the spray tank decreases. The spray pumps are washed after each use to avoid clotting of the insecticide in the nozzles. All spray cans are washed thoroughly, checked, and calibrated once a week. During spraying activities, a quality control officer observes the spraying procedure and technique. Pictures and video recorded randomly by the officer will be used for the quality control evaluation, future reference, or records. Training and evaluation of spray and calibration is provided to all operators every 2 months.

#### Deployment of auto-dissemination devices

Auto-dissemination devices are black plastic containers that mimic breeding sites for female mosquitoes. ADDs use In2Mix powder that contain two active ingredients. The first is technical grade pyriproxyfen (PPF) at doses of 0.375 g per refill sachet. PPF is an insect growth regulator (IGR) that prevents mosquito larvae from developing into adults. The second active ingredient is spores of the insect specific fungus *Beauveria bassiana* GHA strain at 0.05 g per refill sachet. Adult female mosquitoes that visit the ADD pick up the In2Mix powder solution from the statically charged netting. The fungus slowly kills the mosquitoes, while the PPF is transferred by the mosquito to surrounding breeding sites (i.e. auto-dissemination), thereby reducing *Ae. aegypti* populations by decreasing the emergence of new adult mosquitoes [15].

The strategy for ADD deployment in these urban settings was adapted from In2Care standard recommendation (1 trap/400m^2^), based on results from our pilot study in Johor Bahru, Malaysia [21], and another study evaluating the vertical infestation of *Aedes* in high-rise building [22]. Smart deployment of ADDs only on floors with higher expected mosquito density was found to be sufficient to reduce the mosquito population in the whole building.

The exact number of ADDs and suitable location is determined at the start of the study (Additional file 2). ADDs are deployed at the same time as TORS and distributed over each floor as evenly as possible and placed according to the manufacturer’s recommendations in semi-indoor environment such as corridors, multipurpose halls, lobby, prayer hall, shops, and parking (i.e. shaded sites).

#### Community engagement

Community engagement (CE) starts at the time of baseline data collection and is maintained throughout the study period by a team specially trained in CE activities. The main objectives of this activity are to create public awareness and trust and to increase their participation during and following the deployment of the intervention. The first step of CE activities consists of an appointment with the community leaders in each cluster through phone calls using a standard script in order to schedule a face-to-face meeting. The purpose, objectives, procedure, and timelines of the iDEM trial are presented to community leaders followed by a question-and-answer session. Information on demographic and architectural characteristics such as number of buildings, number of floors per building, number of units per floor, total number of occupants, parking lots, and public facilities (gymnasium, kindergarten, shops, restaurants, swimming pool, and playground) of the cluster are recorded after the briefing. A second meeting involving the residents is organized upon the community leader request. Information, education, and communication material such as banners, posters (Additional file 3), and brochures are distributed to explain the objectives of the study and the role of the community during and following the deployment of the intervention. Softcopy of these materials is sent to the building managers through email or WhatsApp message and subsequently disseminated among the community. A notification is sent to the building manager one week before the deployment of the intervention to remind them of the date and details about the intervention.

### Outcomes

The primary outcome measure is the estimated dengue incidence rate in both control and treatment clusters. The number of confirmed dengue cases is provided by the e-Dengue system (http://spwd.arsm.gov.my).

Dengue is a notifiable disease in Malaysia. All clinically suspected or confirmed dengue cases are notified by the medical officer to the nearest District Health Office within 24 h (Additional file 1). Currently, dengue confirmation is based on dengue rapid combo test for (1) qualitative detection and differentiation of specific IgG and IgM antibodies to dengue virus in human serum and plasma and (2) dengue rapid NS1 test for qualitative detection of dengue NS1 antigen in human whole blood, serum, and plasma [23]. Further laboratory tests are performed if necessary (under clinical management not determined by the current trial). The e-Dengue case definition is based on the international classification of diseases ICD (10: A90, A91) and positive diagnostic results of either NS1, IgM, or IgG or any combination of test results [24]. Since 2014, all cases reported in Malaysia are confirmed cases.

Secondary outcomes measures include:

1. Ovitrap index (OI): number of positive ovitraps divided by the total number of recovered ovitraps.
2. Larval index (LI): number of larvae identified divided by the number of ovitraps recovered.
3. Container index (CI): proportion of water-holding surveyed containers infested with *Aedes* larvae or pupae.
4. Adult survey: mean number of female adults *Aedes* collected in sticky ovitraps.
5. Resistance to deltamethrin: measurement of knock-down and mortality rates in wild strain adult mosquito population
6. Residual activity of deltamethrin measured by wall deposit bioassays [25]

### Sample size

The sample size determination was adapted from the bootstrap approach of Kleinman and Huang [26] for cluster randomized trials. To take into account the regression to the mean due to the selection of the localities with the highest incidence rates in 2015–2016, sample size determination was based on the incidence rates of the years 2017–2018. For the purpose of sample size calculation, the estimated number of inhabitants in each cluster was obtained as follows: number of blocks (buildings) x number of floors × number of apartments in each floor × 4.9 (i.e. estimated number of individuals in each apartment is based on National Health and Morbidity Survey 2019 and Department of Statistics). The mean of the cluster-level incidence rates was 0.34% per year (range 0 to 4% per year). The power calculation used resampling with replacement to generate plausible incidence distributions during the future study period, in each arm. The resampling was repeated 5000 times. Each time, the resampled data from the clusters underwent a simulated randomization to the intervention or control arm. For those in the control arm, the inferred dengue incidence rates were calculated from the confirmed dengue cases observed during the years 2017–2018. For clusters in the intervention arm, the inferred incidence rates were calculated from the observed incidence rates multiplied by the expected effect of the intervention. The effect of the intervention was estimated from this simulated dataset using a negative binomial regression model. Finally, the power was estimated as the proportion of analyses with a *p* value ≤ 0.05 (two-sided). If we assume that the minimum effect that we are able to detect is a reduction in the incidence by 33% in the intervention areas (0.34% and 0.23% estimated mean incidence rates per year in the control and intervention arms, respectively), the inclusion of 280 eligible clusters (140 per arm) achieves a power of 85%. This means that, if the rates are as assumed, the study has 85% probability of establishing a benefit of the intervention, using a two-sided significance level of 5%. This power was considered acceptable and this sample size was used for the trial. In order to take into account potential loss of some clusters (refusal to participate, etc…), 300 clusters were randomized in the two arms (150 per arm).

### Allocation

The random allocation of the clusters to the control or the intervention arm (1:1 ratio) was carried out using the PLAN procedure of SAS software, version 9.4 (Copyright (c) 2002-2012 by SAS Institute Inc., Cary, NC, USA), and was stratified on the economic level of the clusters (low or medium) because this is associated with dengue incidence.

In each stratum, clusters were randomly numbered and block randomization were carried out with block sizes of 10 and 16 in order to allocate the clusters to the intervention or control arm. Randomization was carried out after the protocol was approved by the IMR Research Committee.

This trial is not blinded. All clusters were allocated at the start of the trial so an allocation concealment mechanism is not required. Dengue outbreaks are common in Malaysia and controlled by reactive vector control activities. Therefore, discontinuing or modifying the allocated interventions in case of unexpected dengue outbreak was not planned. The allocation sequence has been generated by a statistician of the Service of Biostatistics of Hospices Civils de Lyon, France. The code written by this first statistician was double-checked by a second statistician in regard to the conformity to the randomization protocol. The list of clusters in each arm was saved in a text file and transmitted to the investigators (IMR-Malaysia).

### Data collection

#### Epidemiological data

No active recruitment of dengue cases is planned, as the information on the incidence of dengue during the overall study period in both control and intervention clusters is extracted from the national e-Dengue surveillance database. For each confirmed dengue case, demographic data, signs and symptoms at first medical visit, date of diagnosis, results of the rapid dengue COMBO test, hospitalization, and vital status at discharge are extracted.

#### Entomological data

Entomological data for the assessment of secondary outcomes are collected in 12 clusters randomly selected from each arm (24 in total) and stratified by socio-economic status (medium versus low cost).

#### Climatic parameters

Common climatic parameters (rainfall, temperature, and humidity) have been reported to influence the intensity and magnitude of dengue incidence [27–29]. These parameters will be requested from the Malaysian Meteorological Department, Ministry of Energy, Science, Technology, Environment and Climate Change for two points: Petaling Jaya and Kuala Lumpur International Airport.

### Monitoring *Aedes* population densities

The density of *Aedes aegypti* and *Ae. albopictus* populations will be monitored monthly throughout the study period using larval and adult collection methods in a sample of 12 clusters per arm. Larval surveys are carried out using a minimum of 40 ovitraps per cluster. Each ovitrap consists of a 250-ml cylindrical, black plastic container (7.5 cm diameter, 9.0 cm height, 300 ml volume) filled with tap water to a depth of 5.5 cm and is equipped with a removable oviposition paddle made from a thin strip of brown hardboard (10 cm × 2.5 cm × 0.3 cm). Larval surveys start 5 weeks before the start of the intervention to measure the baseline density. Ovitraps are positioned randomly in spaces with minimum human, physical, and environmental disturbance: (a) in the semi-indoor environment—defined as the area outside of the housing units but still sheltered by the roof (e.g. shared corridor and stairway), (b) in partially or totally shaded outdoor areas (e.g. near bushes, small plants, and temporary structures), and (c) indoor (under the sofa, dining table, sink, bed, working / study desk) only in the apartments of those who volunteer to have them.

To determine the OI, all ovitraps are collected once a week and replaced by new ones with fresh tap water and egg-free oviposition paddles. The collected ovitraps are brought back to the IMR laboratory (Kuala Lumpur) for further processing. Ovitrap contents and oviposition paddles are transferred into plastic containers and labelled with the name of the cluster and date of collection. Larvae emerged from the eggs laid in the ovitraps undergo species identification [30, 31] using a compound microscope (Nikon Eclipse® E100, Japan).

To determine the CI, a minimum of 20 suspected mosquito breeding containers within 50 m radius of the study cluster are examined once a month in each cluster. Larval samples from the positive containers are collected in a sample bottle and brought back for identification. The larvae are allowed to emerge into adults for morphological identification.

To monitor adult mosquito densities, monthly surveys are carried out with sticky ovitraps, i.e. a modification of conventional ovitraps as described by Lau et al [32]. The inner wall of the ovitrap is lined with a 5.5 cm × 24 cm transparent plastic sheet. The inner plastic sheet is covered with insect glue (Neopeace, ACM, Malaysia) attached to the container using adhesive tape on both sides of the top of the container. A hole is drilled about 3 cm above the bottom to avoid flooding of the trap with rain water. A total of 50–200 sticky ovitraps are deployed in the study area, in every alternate floor with emphasis on dark and humid areas such as garbage rooms, emergency exits, shoe racks, and vegetation considered as *Aedes* resting places. The adult samples are isolated in a single vial and send for identification.

### Monitoring mosquito resistance

#### Adult bioassay test

We will measure susceptibility levels to deltamethrin in the field population. The test will follow the WHO protocol [33]. Pyrethroid resistance monitoring is conducted on sugar-fed adult female mosquitoes aged 3 to 5 days against a diagnostic concentration (0.03% deltamethrin, 0.25% permethrin, 0.15% cyfluthrin, and 0.03% lambda-cyhalothrin), which is used to impregnate paper at the recommended exposure time. Five replicates of 20 adult field strain mosquitoes from the 24 clusters selected for entomological endpoints are prepared. Two replicates of susceptible mosquitoes from the insectary laboratory of IMR are used as control strains. The cumulative knockdown counts are recorded every minute within the exposure period of 1 h, or until 90% knockdown is observed. After the exposure period, all the tested mosquitoes (live and knocked down) are transferred into holding tubes for a 24-h recovery period and supplied with 10% sugar solution in cotton balls. Adult mortality is then recorded. If the mortality rate in the control group is between 5 and 20%, the percentage of mortality in the exposed mosquitos is corrected by using Abbot’s formula [33]. Surviving mosquitoes are transferred into Eppendorf tubes and kept at − 80 °C for enzyme micro-assay.

#### Enzyme micro-assay

Three enzyme assays, namely mixed function oxidase [34, 35], esterase [35, 36], and insensitive acetylcholinesterase [35] are conducted in sugar-fed female mosquitoes, aged 3–5 days, to determine the enzyme activity. The female mosquitoes are raised from eggs collected in the field. The same homogenate of an individual mosquito is used to assess all three enzyme assays.

### Monitoring quality of TORS and ADDs post-application

The quality control monitoring for ORS was conducted in 4 clusters using a susceptible laboratory strain mosquito, resistant laboratory strain mosquitoes, and a wild field strain mosquitoes of *Aedes aegypti*. To assess post-application quality of K-Othrine® Polyzone spray, wall deposit bioassays are conducted 48 h after spraying and repeated every 3 weeks for a period of 4 months [25]. Standard WHO bioassay cones are firmly positioned onto the TORS treated wall in a perpendicular position using masking tape. Ten adult female mosquitoes (sucrose-fed, 3–5 days old, non-blood fed) are introduced into the bioassay cone for 30 min through the aperture using a battery-operated aspirator, and then collected back and observed for 24 h mortality. Three biological replicates of 10 mosquitoes are prepared for laboratory and field strains of *Aedes aegypti*. Residual effect of K-Othrine® Polyzone is determined by evaluating the knock down time and mortality rate. Knock down is observed for 30 min at 1-min intervals. After exposure, the mosquitoes are aspirated out, transferred to paper cups, held at 27 ± 2 °C with 75 ± 10% relative humidity and sugar-fed. The mortality is recorded 24 h after testing. The level of resistance is evaluated using the WHO criteria [33].

To assess ADD quality post-application, the presence/absence and estimated quantities of mosquito larvae in the water inside the ADDs are recorded at each service. The water was collected from randomised ADD (3 ADDs) in study sites and tested using laboratory strain of *Aedes aegypti*. Every month, 250 ml water samples are taken from three randomly selected deployed ADDs that are positive for *Aedes* larvae and brought back to the laboratory to confirm the presence and activity of the pyriproxyfen larvicide. In each 250 ml ADD water sample, 25 third instar (L_3_) larvae from the IMR rearing colony of *Aedes aegypti* are added [33]. Emergence of mosquito adults is monitored every day until all larvae have emerged or died or all larvae from control emerge into adult. An emergence rate of adult mosquito less than 10% is indicative of adequate pyriproxyfen quality in the ADD water sample. The samples are collected at the same time as the Container Index sampling and are used for quality control of the ADD and as positive control for the auto-dissemination testing.

To assess auto-dissemination efficacy of the ADDs, water samples of nearby containers are assessed for pyriproxyfen content. The *Aedes*-positive samples from natural containers collected for the CI (as explained above) are used to monitor larval development. Per 10 ml water sample collected, one susceptible strain larvae (L_3_, *Aedes aegypti*) is added to the water. Emergence of adults is monitored every day until all larvae have emerged or died. Significant reductions in adult mosquito emergence rates (i.e. high rates of larval/pupal mortality) in the intervention cluster samples compared to the control cluster samples is indicative of pyriproxyfen autodissemination from the ADDs.

### Data management

Epidemiological data are extracted from the e-Dengue system by the staff of the Vector Borne Disease Sector, Disease Control Division, Ministry of Health Malaysia. Confirmed dengue patients are assigned a unique anonymous code. Entomological data are reported on paper-based forms and subsequently entered into an Excel file by data managers at IMR. A second data manager checks the accuracy of the Excel file by comparing the paper-based forms and data entered in the Excel file. Access to the data is password protected and restricted to authorized study investigators and data management staff.

Both epidemiological and entomological data are sent to the department of Biostatistics in Lyon-France on a monthly basis. Data are controlled for consistency by the team in Lyon. The list of inconsistent or erroneous values (i.e. data queries) is sent to the data managers in IMR for verification. A database in SAS software is prepared for final statistical analyses. A common identifier for clusters is used in order to link the entomological and epidemiological databases.

The original paper-based entomological forms are stored in secured locked cabinets at IMR. As for epidemiological data, access to the data is password protected and restricted to only authorized study investigators and data management staff. The anonymized databases sent to the service of Biostatistics of Hospices Civils de Lyon are stored in the study folder on a secured computer server. Only the biostatisticians in charge of the analyses have access to the data. A daily backup of the study folder is made automatically.

No human samples will be collected for, or held by, this study. Entomology specimens will be stored at IMR. This study will be carried out in compliance with the Malaysian Personal Data Protection Act 2010, stipulating full protection of an individual’s personal information.

All data, documents, the database, and the intervention procedure will be available for audits by regulatory and independent authorities appointed by funders or IMR.

### Plans to promote participant retention and complete follow-up

Our previous study showed that regular contacts between the study population and the field workers create public trust [21]. To maintain adherence to the interventions, a liaison officer assigned to each cluster contacts (via phone call or messaging) the building manager regularly to collect all complaints and feedback from the population. The pest control organisation in charge of TORS and ADDs deployment provides also regularly feed back to the CE team on any concerns faced in the field. These are discussed and taken into consideration in order to improve adherence to the intervention.

### Statistical methods

Statistical analysis will be performed using the statistical analysis software SAS® version 9.4. or higher. Missing data will be reported and their impact on the outcomes may be explored. No interim analyses are planned.

### Primary outcome

The primary endpoint is to measure the effectiveness of the IVM approach on dengue incidence. The mean of the cluster-level incidence rates will be compared between the control and intervention arms. In each cluster, the incidence rate will be estimated as the ratio of the number of dengue cases registered during the trial (starting from the first cycle of spraying and ending three months after the last spraying cycle), divided by the number of person-years. The number of inhabitants per cluster, as obtained for the sample size calculation, is checked and corrected if needed during the site visits for the purpose of calculation of incidence rates.

A negative binomial regression model will be used to compare the incidence between control and intervention clusters as a final value from the end of the first intervention cycle. This will yield the rate ratio and its 95% confidence interval, taking into account overdispersion. The response variable for this analysis will be the number of cases, and the logarithm of the person-years will be included as an offset. Stratification (low versus medium income) will be included as a covariate.

### Secondary outcome

The main secondary outcome will be the OI. The latter will be estimated over the baseline and intervention period in the 24 clusters selected for entomological monitoring. To quantify the effect of the interventions on the proportion of positive ovitraps, a modified ordinary least squares regression model using a robust standard error estimator will be implemented [21]. Using this model, the effect of the interventions will be quantified by the estimation of a difference of risk with its 95% confidence interval. This analysis will be carried out for both species overall and for each of the two species of mosquito separately.

Larval count per ovitrap (LI) will be estimated in the 24 clusters selected for entomological monitoring over the baseline and intervention period. A negative binomial regression will be used with the number of larvae as the response variable. The analysis will be adjusted by the baseline measurement of the outcome. Using this model, the effect of the interventions will be quantified by the estimation of a ratio of means with its 95% confidence interval. This analysis will be carried out for both species overall and for each of the two species of mosquito separately if possible.

The adult population will be estimated by counting the number of adult female *Aedes* mosquitoes collected with sticky ovitraps during the study. The total number of adult females will be divided by the number of sticky ovitraps used to obtain the mean number of adult females.

The emergence rate, defined as the proportion of larvae that emerge as adult mosquitoes, will be estimated in each arm and compared between the two arms using the chi-squared test or the Fisher exact test. This analysis will be carried out for both mosquito species combined and for each species separately.

### Methods in analysis to handle protocol non-adherence and any statistical methods to handle missing data

The main analysis will be by intention to treat. The clusters will be analysed in their randomization arm. As explained above, the primary outcome is provided by the e-Dengue system and will be consequently available for the overall included clusters during the study period. A secondary per protocol analysis will be carried out excluding the clusters with any major deviations to the protocol. Major deviations will be defined by the technical committee (trial governance members) during the data review.

### Trial governance

Implementation of the field work is coordinated by IMR (Malaysia). The Malaysian Ministry of Health provide technical expertise and is in charge of dengue data extraction from the e-Dengue database. The Departments of Epidemiology and Public Health and of Biostatistics (Claude Bernard University and Hospices Civils de Lyon-France) host the project leader and are responsible for statistical analyses. The trial is governed by a technical committee composed of academic/private partnership with skills and knowledge in the epidemiology of infectious disease in particular vector-borne diseases, entomology, new vector control technologies, policy makers, and statistics. The committee defines the study design, review available data, and make decisions on trial conduct.

### Adverse event reporting and harms

The product for spraying outdoor walls is already registered in Malaysia and has been in use for vector control management. Both bio-actives of ADDs have short half-lives and are classified as low risk for non-target organisms. However, instructions about precautionary measures to be taken before and after the intervention are provided in the posters (supplementary material appendix 2) and during community engagement meetings. In addition, a dedicated phone number is provided by the research team to be contacted in the case of an event which might be linked to the interventions. We do not plan to set up a data monitoring committee for the purpose of this cRCT. The trial interventions do not include drugs. On ethical review in Malaysia, the study was assessed as no more than minimal risk.

### Plans for communicating important protocol amendments to relevant parties

Any modification in the protocol that significantly affects the scope or the scientific quality of the investigation will be submitted to the ethical committee with an amendment containing a verbatim description of the changes.

### Dissemination plans

The results of the trial will be disseminated through international scientific conferences and submitted to highly ranked peer-reviewed journals for publication. The findings will also be shared with regional, national, and international stakeholders and partners. Reporting will follow the guidelines in the Consolidated Standards of Reporting Trials (CONSORT) Statement [37]. Authorship will follow guidelines established by the International Committee of Medical Journal Editors (http://www.icmje.org/) which require substantive contributions to the design, conduct, interpretation, and reporting of a trial.

## Discussion

In the absence of vaccines and therapeutics, efficient, sustainable, and cost-effective vector control programmes are considered the principal method of protection against vector-borne diseases. New vector control technologies, prototypes, or products such as spatial repellents, ADDs, sterile insect techniques, and *Wolbachia* are currently under investigation by the Vector Control Advisory Group VCAG-WHO [38]. However, the epidemiological evidence of their impact on the incidence of vector-borne diseases is limited [39].

The iDEM trial aims to address this gap by measuring the effectiveness of a preventive IVM approach on the incidence of dengue. The well-known spatial heterogeneity of dengue incidence that leads to increased baseline inter-cluster variance and the inter-annual fluctuation of dengue epidemic have been taken into consideration during the study power calculation and by conducting the study over 2 years respectively. The results of the trial will provide valuable insights that could be of interest to national and international institutions involved in the control of dengue.

The vector control tools of the proposed IVM are relatively new and have been scientifically proven to be effective against vectors [3, 21, 40]. High bio-efficacy for K-Othrine® Polyzone used in semi-indoor areas with > 80% mortality for a period of almost 4 months has been reported in two studies carried out in Malaysia [21, 41]. These results suggest that TORS can potentially reduce the frequency of current insecticide applications, in particular in densely populated urban districts where coverage of indoor preventive measures is low. ADDs are currently registered and used in over 45 countries worldwide and were shown to be effective as auto-dissemination device under field conditions [21, 40].

The choice of RCT was based on the WHO guideline that considers this type of trial as level 1 of the hierarchy of study designs that is sufficiently robust to generate evidence on the efficacy and public health value of vector control products [12]. The cluster rather than individual-based RCT is well adapted to assess organizational and behavioural interventions, health promotion programs, or interventions that are usually implemented at the level of health organizational units or geographical areas. Although such design requires large sample size, it reduces contamination between those assigned to the intervention or control arm. The large scale cRCT described in this protocol is unique in its approach. It combines two relatively new control tools that complement each other by targeting both larval and adult stages of the mosquito’s life cycle. Using this combination, the TORS method will potentially reduce the infective adult mosquito while the ADDs will help to sustain the mosquito population below the threshold as described in the Malaysian Ministry of Health guideline [42]. This is further complemented by extensive public engagement activities to make the public better aware of the problems and solutions, encouraging a behavioural change. This could potentially increase the efficacy of vector control programmes and decrease dengue incidence.

The study area is suitable for the proposed trial because Malaysia is one of the Asian countries hardest hit by dengue, with all its current 32.6 million inhabitants at risk. The country has appropriate infrastructure (expertise in vector control management, strong social mobilization capacities, and existence of surveillance systems) that facilitates the successful implementation of such large-scale trials. The WHO-IVM approach [13] underlines the importance of strengthening communication among different healthcare bodies such as policy-makers and vector-borne disease programme managers for the implementation of mosquito control strategies. The trial is, therefore, carried out in close collaboration with the Malaysian Ministry of Health, enabling local support and continuity of the proposed IVM approach if promising results are observed.

The trial is designed to measure the reduction in the incidence of dengue in the intervention relative to the control areas using incidence data extracted from the national e-Dengue surveillance system. Although passive dengue surveillance in endemic countries is essential for timely detection and containment of outbreaks, it is subject to under-ascertainment (cases not being diagnosed) and under-reporting (cases being diagnosed but not included in surveillance data). In Malaysia, failure to report leads to penalties for medical doctors. This is therefore likely to reduce the under-reporting, in particular for symptomatic dengue cases. Imperfection of the dengue diagnosis tests is another concern [43]. The use of combined IgM and NS1 detection into one test in Malaysia allows for high sensitivity during both the early (NS1) and late (IgM) phases of the disease, thus improving the overall test performance. Only cases with positive RDT test and fulfilling the international clinical criteria (ICD10: A90, A91) are registered in the e-Dengue surveillance system, thereby improving the specificity of the system [23].

One possible source of bias is that due to the nature of the intervention, blinding was not feasible. A feeling of protection provided by the intervention could potentially affect health-seeking behaviour in case of a febrile episode or to a lesser extent to the use of other preventive measures. The latter reasoning has been suggested as an explanation in a recent study that evaluated the impact of insecticide treated curtains on dengue virus transmission [44]. Another concern is the continued compliance of participating clusters and their collaboration with the field workers during the intervention cycles to remove bulky objects from the corridors at the time of TORS spraying. Public trust in the Malaysian Ministry of Health, responsible for routine dengue prevention and control, and representing the main coordinator of the present intervention together with continuous dialogue with the community by providing information about the programme is expected to alleviate this issue. The routine vector control activities, which clearly are not being interrupted, will be monitored. However, these interventions would cause only an underestimation of the true effect of the proposed IVM strategy, relative to no action.

Even if efficient in the prevention and control of dengue, particularly for difficult to treat urban setting, a vector control programme such as the preventive IVM approach presented here can be scaled-up as a national programme if it is cost effective. Analysis of the cost-effectiveness of the proposed IVM strategy compared to the routine vector control activities is therefore a key parameter for its future adaptation as a national vector control program. Efforts are ongoing to secure the budget for the economic evaluation of our trial that will be conducted as an ancillary study of the present protocol.

In conclusion, a long-term residual effect of K-Othrine® Polyzone [21, 41], proven efficacy of ADDs [15, 16, 40], and relatively simple maintenance of both vector control methods would enable a sustainable preventative strategy instead of a responsive vector control. We expect that co-application of these vector control tools together with public cooperation could prove an invaluable vector control approach to reduce the burden of dengue. By regular communication with the residents, we hope to create local capabilities by transferring responsibilities (e.g. adding water to dried ADDs) and promote their participation (e.g. destruction of breeding sites) in national efforts to reduce the burden of dengue.

Considering the ongoing expansion of dengue burden in Malaysia, setting up proactive vector control strategies are critical. The results will be informative for a better understanding of effectiveness of proactive IVM approach in the control of dengue. Evidence from this trial may help justify investment in preventive IVM approaches as preferred to reactive case management strategies. It is conceivable that the result of this trial could be used in other locations with similar ecology or adapted to other *Aedes* borne diseases with same vector and process of transmission.

## Supplementary Information

**Additional file 1.** Flowchart of notification and registration of dengue cases in e-Dengue surveillance system

**Additional file 2.** Strategy for deployment of Auto-dissemination Devices

**Additional file 3.** Example of communication material: Poster installed in all intervention areas summarizing the intervention procedure and rules

**Additional file 4.** SPIRIT 2013 Checklist: Recommended items to address in a clinical trial protocol and related documents*

## Data Availability

Source documents as well as entomological specimens will be stored at the Medical Entomology Unit, Infectious Diseases Research Centre, Institute for Medical Research (Kuala Lumpur-Malaysia). The final dataset generated for the final analysis will be stored in the department of Biostatistics, Hospices Civils de Lyon-France. The database that will support the findings of the study will be available from the authors upon reasonable request and with permission of the Malaysian Ministry of Health. The full protocol and statistical code will be accessible upon request. Participant level-data are not expected to be available.

## Abbreviations

ADD: Auto-dissemination device
CE: Community engagement
cRCT: Cluster randomized controlled trials
CI: Container Index
ICD: International classification of diseases
IgG: Immunoglobulin G
IgM: Immunoglobulin M
IGR: Insect growth factor
iDEM: Intervention for Dengue Epidemiology in Malaysia
IMR: Institute for Medical Research
IVM: Integrated vector management
LI: Larval Index
OI: Ovitrap Index
PPF: Pyriproxyfen
TORS: Targeted outdoor residual spray
WHO: World Health Organization
