# Supplementary material for: Measuring the effectiveness of integrated vector management with targeted outdoor residual spraying and autodissemination devices on the incidence of dengue in urban Malaysia in the iDEM trial (intervention for Dengue Epidemiology in Malaysia): study protocol for a cluster randomized controlled trial
Source: Trials. 2021 May 30;22:374. doi: 10.1186/s13063-021-05298-2 (PMC8166066; doi:10.1186/s13063-021-05298-2)
Supplement: Supplementary file 1 — Additional file 1. Flowchart of notification and registration of dengue cases in e-Dengue surveillance system [file 13063_2021_5298_MOESM1_ESM.docx]

**Additional file 1**

**Flowchart of notification and registration of dengue cases in e-Dengue surveillance system**

All clinically suspected or laboratory confirmed dengue cases are notified by the medical officers and registered in the eNotification system within 24 hours. Confirmed dengue cases are then recorded in the eDengue system within 24 hours from the date of notification. The state health office informs the vector borne disease sector of the Ministry of Health to set-up responsive vector control activities in the area where the case had occurred.
