# Supplementary material for: Measuring the effectiveness of integrated vector management with targeted outdoor residual spraying and autodissemination devices on the incidence of dengue in urban Malaysia in the iDEM trial (intervention for Dengue Epidemiology in Malaysia): study protocol for a cluster randomized controlled trial
Source: Trials. 2021 May 30;22:374. doi: 10.1186/s13063-021-05298-2 (PMC8166066; doi:10.1186/s13063-021-05298-2)
Supplement: Supplementary file 2 — Additional file 2. Strategy for deployment of Auto-dissemination Devices [file 13063_2021_5298_MOESM2_ESM.docx]

**Additional file 2**

**Strategy for deployment of Auto-dissemination Devices**

The strategy for ADD deployment in high-rise buildings in urban settings was adapted from the manufacturers recommendation (1 ADD/400m^2^), considering results from studies carried out in Malaysia [Hamid et al. 2020a; Hamid et al. 2020b]. ADDS will be deployed in the ground floors, first floors, top floors and evenly among intermediate floors (Figure 1).

*Ground floor*

The ground floor is defined as the floor at the same level of the ground and/or with access to the street. Ground floor shall consist of either one or combination of these: lobby, parking area, living unit, and public facilities for residents (office, shops, swimming pool, garden, park, playground, multipurpose hall, prayer hall and etc.). The first floor is defined as the floor of a building that is above the ground floor and consists of either living unit only or combination of parking area, living unit, and public facilities for residents. These floors have in general high density of mosquito breeding [Hamid et al. 2020b]. In localities (clusters) with more than one entrance per building, each entrance will be treated as one block in the ADD standard calculation method.

Calculation of the number of ADDs in the ground floors imply only the semi-indoor areas. The number of ADDs required for deployment in the outdoor area of the locality is calculated according to the manufacturers recommendation of 1 ADD per 400m^2^. The estimated number of ADDs from the calculation is rounded up. ADDs will be evenly distributed over the ground floor areas and placed according to the manufacturer’s recommendations using the equation below.

$$Number of ADDs for the ground floor=\frac{Number of living units on an average floor}{4}$$

*Parking floor*

For parking floor either at basement, ground floor or any floor above ground floor, one ADD will be deployed either at the lift lobby, exit door, or entrance to the parking.

*General floors*

The number of ADDs required for each floor depends on the number of living units per floor as indicated below. ADDs will be evenly distributed over a floor and placed according to the manufacturer’s recommendations.

$$Number of ADDs for a general floor=\frac{Number of living units on an average floor}{8}$$

|  |  | Number of floors | | | | | | | | | | | | | | | | | | | | | | |
| --- | --- | --- | --- | --- | --- | --- | --- | --- | --- | --- | --- | --- | --- | --- | --- | --- | --- | --- | --- | --- | --- | --- | --- | --- |
| Number of floors | Status | 5 | 6 | 7 | 8 | 9 | 10 | 11 | 12 | 13 | 14 | 15 | 16 | 17 | 18 | 19 | 20 | 21 | 22 | 23 | 26 | 30 | 35 | 39 |
| 38 | Living Unit |  |  |  |  |  |  |  |  |  |  |  |  |  |  |  |  |  |  |  |  |  |  |  |
| 37 | Living Unit |  |  |  |  |  |  |  |  |  |  |  |  |  |  |  |  |  |  |  |  |  |  |  |
| 36 | Living Unit |  |  |  |  |  |  |  |  |  |  |  |  |  |  |  |  |  |  |  |  |  |  |  |
| 35 | Living Unit |  |  |  |  |  |  |  |  |  |  |  |  |  |  |  |  |  |  |  |  |  |  |  |
| 34 | Living Unit |  |  |  |  |  |  |  |  |  |  |  |  |  |  |  |  |  |  |  |  |  |  |  |
| 33 | Living Unit |  |  |  |  |  |  |  |  |  |  |  |  |  |  |  |  |  |  |  |  |  |  |  |
| 32 | Living Unit |  |  |  |  |  |  |  |  |  |  |  |  |  |  |  |  |  |  |  |  |  |  |  |
| 31 | Living Unit |  |  |  |  |  |  |  |  |  |  |  |  |  |  |  |  |  |  |  |  |  |  |  |
| 30 | Living Unit |  |  |  |  |  |  |  |  |  |  |  |  |  |  |  |  |  |  |  |  |  |  |  |
| 29 | Living Unit |  |  |  |  |  |  |  |  |  |  |  |  |  |  |  |  |  |  |  |  |  |  |  |
| 28 | Living Unit |  |  |  |  |  |  |  |  |  |  |  |  |  |  |  |  |  |  |  |  |  |  |  |
| 27 | Living Unit |  |  |  |  |  |  |  |  |  |  |  |  |  |  |  |  |  |  |  |  |  |  |  |
| 26 | Living Unit |  |  |  |  |  |  |  |  |  |  |  |  |  |  |  |  |  |  |  |  |  |  |  |
| 25 | Living Unit |  |  |  |  |  |  |  |  |  |  |  |  |  |  |  |  |  |  |  |  |  |  |  |
| 24 | Living Unit |  |  |  |  |  |  |  |  |  |  |  |  |  |  |  |  |  |  |  |  |  |  |  |
| 23 | Living Unit |  |  |  |  |  |  |  |  |  |  |  |  |  |  |  |  |  |  |  |  |  |  |  |
| 22 | Living Unit |  |  |  |  |  |  |  |  |  |  |  |  |  |  |  |  |  |  |  |  |  |  |  |
| 21 | Living Unit |  |  |  |  |  |  |  |  |  |  |  |  |  |  |  |  |  |  |  |  |  |  |  |
| 20 | Living Unit |  |  |  |  |  |  |  |  |  |  |  |  |  |  |  |  |  |  |  |  |  |  |  |
| 19 | Living Unit |  |  |  |  |  |  |  |  |  |  |  |  |  |  |  |  |  |  |  |  |  |  |  |
| 18 | Living Unit |  |  |  |  |  |  |  |  |  |  |  |  |  |  |  |  |  |  |  |  |  |  |  |
| 17 | Living Unit |  |  |  |  |  |  |  |  |  |  |  |  |  |  |  |  |  |  |  |  |  |  |  |
| 16 | Living Unit |  |  |  |  |  |  |  |  |  |  |  |  |  |  |  |  |  |  |  |  |  |  |  |
| 15 | Living Unit |  |  |  |  |  |  |  |  |  |  |  |  |  |  |  |  |  |  |  |  |  |  |  |
| 14 | Living Unit |  |  |  |  |  |  |  |  |  |  |  |  |  |  |  |  |  |  |  |  |  |  |  |
| 13 | Living Unit |  |  |  |  |  |  |  |  |  |  |  |  |  |  |  |  |  |  |  |  |  |  |  |
| 12 | Living Unit |  |  |  |  |  |  |  |  |  |  |  |  |  |  |  |  |  |  |  |  |  |  |  |
| 11 | Living Unit |  |  |  |  |  |  |  |  |  |  |  |  |  |  |  |  |  |  |  |  |  |  |  |
| 10 | Living Unit |  |  |  |  |  |  |  |  |  |  |  |  |  |  |  |  |  |  |  |  |  |  |  |
| 9 | Living Unit |  |  |  |  |  |  |  |  |  |  |  |  |  |  |  |  |  |  |  |  |  |  |  |
| 8 | Living Unit |  |  |  |  |  |  |  |  |  |  |  |  |  |  |  |  |  |  |  |  |  |  |  |
| 7 | Living Unit |  |  |  |  |  |  |  |  |  |  |  |  |  |  |  |  |  |  |  |  |  |  |  |
| 6 | Living Unit |  |  |  |  |  |  |  |  |  |  |  |  |  |  |  |  |  |  |  |  |  |  |  |
| 5 | Living Unit |  |  |  |  |  |  |  |  |  |  |  |  |  |  |  |  |  |  |  |  |  |  |  |
| 4 | Living Unit |  |  |  |  |  |  |  |  |  |  |  |  |  |  |  |  |  |  |  |  |  |  |  |
| 3 | Living Unit |  |  |  |  |  |  |  |  |  |  |  |  |  |  |  |  |  |  |  |  |  |  |  |
| 2 | Living Unit |  |  |  |  |  |  |  |  |  |  |  |  |  |  |  |  |  |  |  |  |  |  |  |
| 1 | Living Unit |  |  |  |  |  |  |  |  |  |  |  |  |  |  |  |  |  |  |  |  |  |  |  |
| G | Ground Floor |  |  |  |  |  |  |  |  |  |  |  |  |  |  |  |  |  |  |  |  |  |  |  |
|  |  |  |  |  |  |  |  |  |  |  |  |  |  |  |  |  |  |  |  |  |  |  |  |  |

**Figure 1: Distribution of ADDs in different floors (in blue) based on the number of floors per building**

**ADD Set Up**

ADDs will be equipped with 3 stickers (Figure 2): 1) on the lid that contains the date of deployment and the ADD’s number, 2) on the container explaining the function of the ADD, and 3) on the wall to inform the community about the placement of the ADDs. The operators are given a diagram on the location and number of ADDs to set up. ADDs are tied with cable tie and the location is recorded in a record book. The recording number includes the locality, block, floor and the ADD number.

**
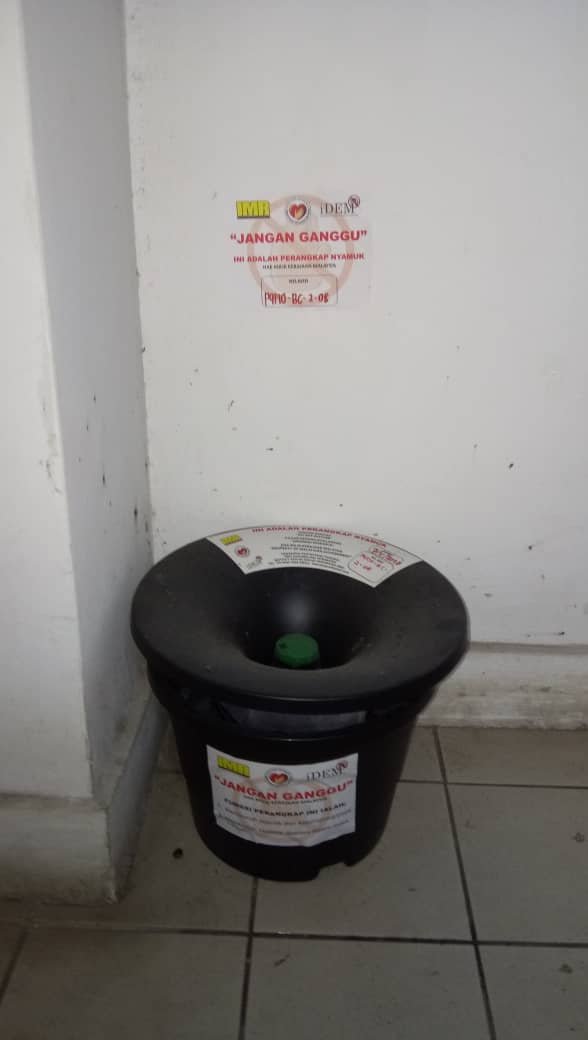
**

**Figure 2: Example of ADD set-up with stickers**

**ADD monitoring and Servicing**

A monitoring checklist form is provided to operators to collect information on ADD status (see ADD monitoring form below). Deployed ADDs must have a lid, floater, netting, and container. ADDs will be serviced every 8 weeks to ensure that there is still adequate amount of the active ingredients, to clean up the ADDs if needed, and to top-up the water inside the container. Incomplete or damaged ADDs will be recorded. Any missing or damaged parts are replaced at the time of servicing.


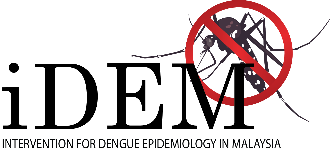


**ADD MONITORING FORM**

| **OPERATOR’S NAMES** | | | **1)** | | | | | | | **QC PERSONNEL** | | |  | | | | | | | |
| --- | --- | --- | --- | --- | --- | --- | --- | --- | --- | --- | --- | --- | --- | --- | --- | --- | --- | --- | --- | --- |
|  |  |  | **2)** | | | | | | | **DATE** | | |  | | | | | | | |
|  | | | | | | | | | | | | | | | | | |  |  | |
| **NO ADD** | **ADD CONDITION** | | | | | | | | | | **LARVAL NO** | | | **ADD SERVICE** | | | | | | |
|  | **COMPLETE SET** | **MISSING COVER** | | **MISSING FLOATER** | **MISSING NET** | **MISSING BUCKET** | **MISSING ADD** | **DRY WATER** | **DIRTY WATER** | **RUBBISH** | **<50** | **>50** | | **REPLAVE COMPLE SET** | **REPLACE MISSING PART** | **ADD IN2MIX** | **ADD WATER** | **ADD REMOVED** | **RELOCATE ADD** |  |
|  |  |  | |  |  |  |  |  |  |  |  |  | |  |  |  |  |  |  |  |
|  |  |  | |  |  |  |  |  |  |  |  |  | |  |  |  |  |  |  |  |
|  |  |  | |  |  |  |  |  |  |  |  |  | |  |  |  |  |  |  |  |
|  |  |  | |  |  |  |  |  |  |  |  |  | |  |  |  |  |  |  |  |
|  |  |  | |  |  |  |  |  |  |  |  |  | |  |  |  |  |  |  |  |
|  |  |  | |  |  |  |  |  |  |  |  |  | |  |  |  |  |  |  |  |
|  |  |  | |  |  |  |  |  |  |  |  |  | |  |  |  |  |  |  |  |
|  |  |  | |  |  |  |  |  |  |  |  |  | |  |  |  |  |  |  |  |
|  |  |  | |  |  |  |  |  |  |  |  |  | |  |  |  |  |  |  |  |
