# Supplementary material for: Measuring the effectiveness of integrated vector management with targeted outdoor residual spraying and autodissemination devices on the incidence of dengue in urban Malaysia in the iDEM trial (intervention for Dengue Epidemiology in Malaysia): study protocol for a cluster randomized controlled trial
Source: Trials. 2021 May 30;22:374. doi: 10.1186/s13063-021-05298-2 (PMC8166066; doi:10.1186/s13063-021-05298-2)
Supplement: Supplementary file 3 — Additional file 3. Example of communication material: Poster installed in all intervention areas summarizing the intervention procedure and rules [file 13063_2021_5298_MOESM3_ESM.docx]

**Additional file 3**

**Example of communication material: Poster installed in all intervention areas summarizing the intervention procedure and rules**
